# Passive Self‐Sustained Thermoelectric Devices Powering the 24 h Wireless Transmission via Radiation‐Cooling and Selective Photothermal Conversion

**DOI:** 10.1002/advs.202309871

**Published:** 2024-04-04

**Authors:** Kai Liu, Yaoguang Ma, Yuzheng Li, Yunxiao Wu, Chenguang Fu, Tiejun Zhu

**Affiliations:** ^1^ State Key Laboratory of Silicon and Advanced Semiconductor Materials and School of Materials Science and Engineering Zhejiang University Hangzhou 310058 China; ^2^ Shanxi‐Zheda Institute of Advanced Materials and Chemical Engineering Taiyuan 030000 China; ^3^ State Key Laboratory for Extreme Photonics and Instrumentation College of Optical Science and Engineering Intelligent Optics and Photonics Research Center Jiaxing Research Institute Zhejiang University Hangzhou 310058 China; ^4^ ZJU‐Hangzhou Global Scientific and Technological Innovation Center Zhejiang University Hangzhou 310058 China

**Keywords:** radiation cooling, selective photothermal absorption, self‐sustained power supply, thermoelectric device

## Abstract

The rapid development of the Internet of Things has triggered a huge demand for self‐sustained technology that can provide a continuous electricity supply for low‐power electronics. Here, a self‐sustained power supply solution is demonstrated that can produce a 24 h continuous and unipolar electricity output based on thermoelectric devices by harvesting the environmental temperature difference, which is ingeniously established utilizing radiation cooling and selective photothermal conversion. The developed prototype system can stably maintain a large temperature difference of about 1.8 K for a full day despite the real‐time changes in environmental temperature and solar radiation, thereby driving continuous electricity output using the built‐in thermoelectric device. Specifically, the large output voltage of >102 mV and the power density of >4.4 mW m^−2^ could be achieved for a full day, which are outstanding among the 24 h self‐sustained thermoelectric devices and far higher than the start‐up values of the wireless temperature sensor and also the light‐emitting diode, enabling the 24 h remote data transmission and lighting, respectively. This work highlights the application prospects of self‐sustained thermoelectric devices for low‐power electronics.

## Graphical abstract

Here, a 24 h self‐sustained power supply solution using thermoelectric technology is demonstrated by harvesting the environmental continuous and unipolar temperature difference, which is ingeniously established utilizing the radiation‐cooling effect and selective photothermal conversion. The developed prototype system can stably support the 24 h remote data collection by powering the wireless temperature sensors and light‐emitting diode (LED) lighting.

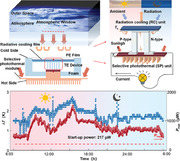

## 1 Introduction

The worldwide number of Internet of Things (IoT) devices is forecast to almost triple from 9.7 billion in 2020 to more than 29 billion in 2030.^[^ ^1^, ^2^ ^]^ Entering the era of IoT, there is a surging growth of low‐power electronics for information acquisition and transmission applications, triggering a significant demand for self‐sustained power supply technology. Currently, disposable batteries and/or rechargeable batteries are widely used for the power supply of low‐power IoT nodes.^[^ ^3^, ^4^ ^]^ However, the ten‐billion‐level IoT nodes will generate equal or more battery consumption, which is inconsistent with the pursuit of a low‐carbon economy. For some scenes that are sparsely populated or environmentally atrocious, the cost of battery supplement, replacement, and recovery is very high. As the Internet of Things (IoT) finds its way into various industries, conventional wired power supplies and batteries are facing significant challenges in meeting the demands for cost‐efficiency, energy conservation, and environmental protection. This drives the development of self‐sustained power supply technology.

Passive energy conversion technologies, including photovoltaics,^[^ ^5^, ^6^ ^]^ triboelectrics,^[^ ^7^, ^8^ ^]^ piezoelectrics,^[^ ^9^, ^10^ ^]^ magnetoelectrics,^[^ ^11^, ^12^ ^]^ and radio frequency,^[^ ^13^ ^]^ can convert environmentally distributed energy into electricity, providing potential solutions for the self‐sustained supply, but there are still limitations. For instance, silicon solar cells have achieved a very high conversion efficiency of 26.7%.^[^ ^14^, ^15^ ^]^ However, they are unable to generate electricity at night due to the lack of light. For triboelectric and piezoelectrics, electrical energy can only be generated in situations where there is mechanical energy such as human motion and wind.^[^ ^16^, ^17^ ^]^ Magnetoelectric and radio frequency conversion require high‐intensity magnetic fields to achieve normal power supply applications.^[^ ^18^ ^]^ As another blue‐chip technology, solid‐state thermoelectric (TE) technology can convert ubiquitous heat into electricity as long as a temperature difference (Δ*T*) across the built‐in TE device can be maintained.^[^ ^19^, ^20^ ^]^ This is because the charge carriers in the thermoelectric materials will undergo directional movement under the applied Δ*T* when connecting with an external circuit. Specifically, as long as Δ*T* is kept stable over time, for instance, by utilizing solar energy,^[^ ^21^, ^22^ ^]^ human skin surface heat,^[^ ^23^, ^24^ ^]^ or waste heat,^[^ ^25^ ^]^ the TE device can generate continuous and stable electricity output over time. Given its promising applications in diverse fields like the IoT and wearable/flexible electronics, TE conversion technology has garnered increasing research attention in recent years.^[^ ^26^, ^27^ ^]^ Nevertheless, several important challenges hinder the applications of TE technology. One of them is how to achieve a self‐sustained and stable Δ*T* across the built‐in TE device over time.

Since nighttime radiation‐cooling performance was improved by a selective infrared emitter in 1959,^[^ ^28^ ^]^ passive radiation‐cooling (PRC) materials with large long‐wave infrared (LWIR) emissivity or absorptivity (${\bar{\varepsilon }}_{\mathrm{LWIR}}$) in the atmospheric window (8–13 µm) and solar reflectivity (${\bar{R}}_{\mathrm{solar}}$) in the solar spectrum (0–2.5 µm) have attracted extensive research concerns.^[^ ^29^, ^30^ ^]^ Utilizing the huge Δ*T* between the Earth (≈300 K) and outer space (≈3 K), PRC materials can transfer heat to outer space owing to their high ${\bar{\varepsilon }}_{\mathrm{LWIR}}$ and ${\bar{R}}_{\mathrm{solar}}$, thereby their temperature can be lower than the environment without external intervention. A maximal temperature drop of 42 K was achieved by reducing the parasitic thermal load.^[^ ^31^ ^]^ A hierarchically porous poly(vinylidene fluoride‐co‐hexafluoropropene) [P(VdF‐HFP)_HP_] film with high PRC ability was reported to generate sub‐environmental temperature drops of about 6 K and cooling powers of 96 W m^−2^ under solar radiation intensities of 890 and 750 W m^−2^, respectively.^[^ ^32^ ^]^ Glass‐polymer hybrid metamaterials backed with the silver coating showed an average radiation‐cooling power of >110 W m^−2^ over the continuous day‐and‐night test for 72 h.^[^ ^33^ ^]^ Therefore, PRC materials can form a sustained temperature difference with the environment, which may be utilized by TE devices.

Recently, passive self‐sustained thermoelectric (PSS‐TE) devices based on the radiation‐cooling effect have attracted increasing attention. In 2019, a PSS‐TE device was proposed by Fan et al.,^[^ ^34^ ^]^ of which the cold end could radiate heat to the cold of space, enabling continuous power generation and light‐emitting diode (LED) lighting at night, marking the pioneering research of PSS‐TE devices. Subsequently, the PSS‐TE device was improved by optimizing size parameters, material properties,^[^ ^35^, ^36^, ^37^ ^]^ and structure design.^[^ ^38^, ^39^ ^]^ Despite these successful attempts, the electricity output of current PSS‐TE devices still has a large room to improve. Besides, another common challenge is that solar irradiation in the daytime will weaken the radiation‐cooling effect, leading to a significantly reduced Δ*T*.^[^ ^40^, ^41^, ^42^ ^]^ To address this challenge, the moisture‐induced,^[^ ^43^ ^]^ photothermal,^[^ ^44^, ^45^, ^46^ ^]^ or greenhouse^[^ ^47^, ^48^ ^]^ effects have been proposed to maintain the Δ*T* by increasing the temperature on the hot end of the TE device. However, the mismatch between the two working mechanisms, that is, photothermal materials on the hot end can also have a radiation‐cooling effect at night while radiation‐cooling materials on the cold end produce a photothermal effect in the daytime, will cause a large fluctuation or reversal in Δ*T* and thus unstable electricity output for a full‐day application. Achieving a 24 h continuous and practicable Δ*T* (>1 K) for PSS‐TE devices throughout the day and night is still an important challenge, which, if well solved, will significantly advance the practical application of this technology. Seeking new strategies to achieve a good match of multiple energy conversion mechanisms to fully exert their effects both in the daytime and at night is important for improving the Δ*T* and maintaining 24 h stability. The solution to this challenge will play a crucial role in fostering the advancement of PSS‐TE technology for the long‐term self‐sustained power supply.

Here, a 24 h self‐sustained power supply solution using the TE technology has been demonstrated by harvesting the environmental continuous and unipolar Δ*T*, which is ingeniously established utilizing the radiation‐cooling effect and selective photothermal conversion through spectrum coupling and materials regulation. A continuous and practicable electricity output that is excellent among the 24 h self‐sustained TE devices was achieved, which takes the lead to support the 24 h remote data collection by powering the wireless temperature sensors and LED lighting. These results provide a solution for the practical application of PSS‐TE technology for powering low‐power electronics in the field of IoT.

## 2 Design of 24 h PSS‐TE System

**Figure 1:**
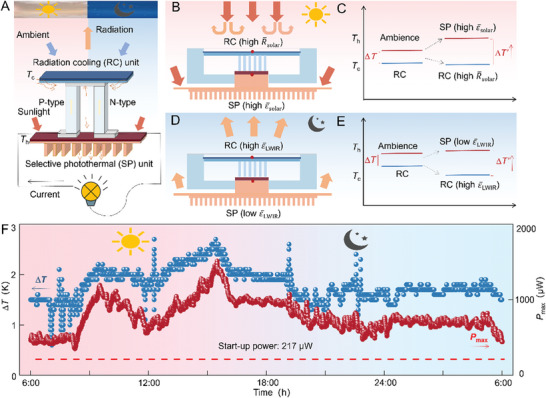
Schematic working mechanism and real‐time electric output for the developed 24 h PSS‐TE device. A) The radiation–cooling and selective photothermal form cold and hot ends at both ends of the thermoelectric device, respectively. B,C) The selective photothermal plays a major role in establishing Δ*T* in the day. D,E) The radiation cooling plays a major role in establishing Δ*T* at night. F) A continuous and unipolar 24 h Δ*T* and electricity output power *P*_max_ is generated by the PSS‐TE device. The start‐up power of the employed commercial wireless temperature sensor is 217 µW.

As discussed above, the key to achieving a continuous and practicable 24 h electricity output for the PSS‐TE system lies in the establishment of a continuous and unipolar Δ*T* at both ends of the built‐in TE device. For some existing PSS‐TE devices, the Δ*T* at night is created based on the radiation‐cooling effect on the cold end of the TE device. In the daytime, solar irradiation will weaken the radiation‐cooling effect and thus significantly reduce or even reverse the Δ*T*. To maintain the unipolar and stable Δ*T*, coating the photothermal materials on the hot end of the TE device to increase its temperature is a potential solution. However, the mismatch of spectral characteristics between the radiation‐cooling film (poor solar reflection ability) and the photothermal film (high radiation‐cooling effect) would cause the largely fluctuated or even reversed Δ*T*, which cannot guarantee a stable or unipolar electricity output. To overcome this problem, this work achieves a 24 h continuous and unipolar Δ*T* by coupling selective photothermal absorption and radiation cooling based on spectral characteristics. The schematic working principle of the developed PSS‐TE device is shown in **Figure** **1**A (details can be found in Note S1, Supporting Information). In the daytime, the selective photothermal film with high solar emissivity or absorptivity (${\bar{\varepsilon }}_{\mathrm{solar}}=\;1-{\bar{R}}_{\mathrm{solar}}$) in the solar spectrum (0–2.5 µm) absorbs sunlight and forms the hot end while the radiation‐cooling film with a high ${\bar{R}}_{\mathrm{solar}}$ forms a cold end by reducing the deposition of sunlight (Figure 1B,C). At night, the radiation‐cooling film forms a cold end due to its high ${\bar{\varepsilon }}_{\mathrm{LWIR}}$ by radiating the heat to outer space, while the selective photothermal film with a low ${\bar{\varepsilon }}_{\mathrm{LWIR}}$ can reduce its heat radiation and the heat convection occurs between the heat sink and the environment, thus forming a hot end (Figure 1D,E).

Owing to the coupling mechanism of the radiation‐cooling film and the selective photothermal film, a continuous self‐sustained Δ*T* with an average value of 1.8 K for the whole day was successively achieved in the prototype PSS‐TE device. As a result, a 24 h electricity output higher than 376 µW was achieved (Figure 1F). The PSS‐TE device can successfully drive a commercial wireless temperature sensor which has a startup power of 217 µW to continuously transmit wireless temperature signals for 24 h (to be shown later). These results demonstrate the application prospect of the developed PSS‐TE device, which is capable of generating electricity continuously and stably, powering IoT nodes from day to night.

## 3 Results and Discussion

### 3.1 COMSOL Simulation

**Figure 2:**
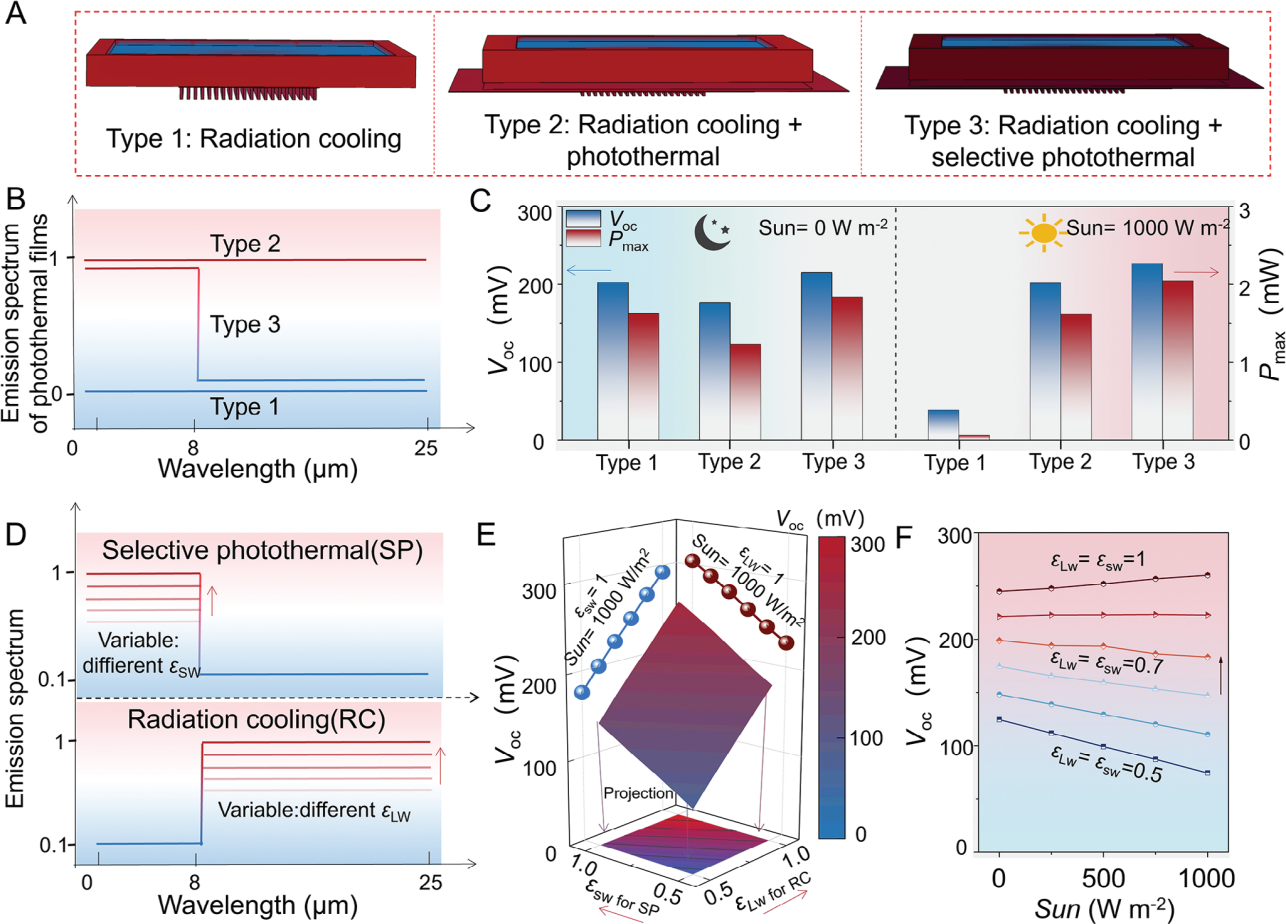
COMSOL simulation analysis. A) Structural diagram of three PSS‐TE devices. B) The emission spectrum of photothermal films in the hot end of PSS‐TE devices. C) The *V*_oc_ and *P*_max_ of three PSS‐TE devices at night and in the daytime, respectively. D) Emission spectrum of the radiation‐cooling film with different *ε*_LW_ and the selective photothermal film with different *ε*_SW_. E) The mapped *V*_oc_ under different *ε*_LW_ and *ε*_SW_. F) The mapped *V*_oc_ under different solar radiation densities.

Although the combination of radiation cooling and photothermal effects has been reported in TE devices,^[^ ^44^, ^45^, ^46^ ^]^ the response relationship between their spectral characteristics and continuous unipolar electrical output is not clear. To examine the coupling mechanism of radiation cooling and selective photothermal for achieving the 24 h electricity output, three PSS‐TE devices, named Type 1, Type 2, and Type 3, with different mechanisms, were first studied using the COMSOL simulation (**Figure** **2**A). Relevant materials and dimensional parameters used in the simulation are shown in Table S1 (Supporting Information) (Note S2, Supporting Information). Type 1 merely utilizes the radiation‐cooling effect to form a Δ*T* with the environment. Type 2 couples the radiation‐cooling effect with the photothermal effect to form a Δ*T*, where the employed photothermal material is blackbody‐like and has high absorptivity or emissivity in the full‐spectrum range (0–25 µm). Type 3 contains both radiation‐cooling and selective photothermal effects, in which the selective photothermal material has both high ${\bar{\varepsilon }}_{\mathrm{solar}}$ and low ${\bar{\varepsilon }}_{\mathrm{LWIR}}$. The emission spectrum of photothermal films in the hot end of PSS‐TE devices (Figure 2B) shows the difference between the three types.

The open‐circuit voltage (*V*_oc_) and maximum output power (*P*_max_) of the PSS‐TE device at night (i.e., the solar irradiation intensity Sun = 0 W m^−2^) and in the daytime (Sun = 1000 W m^−2^) are shown in Figure 2C. The electricity output of Type 1 in the daytime drops significantly compared to the night, because the solar radiation deposited on the surface of the radiation‐cooling film will increase the temperature of the cold end in the daytime and offset the radiation‐cooling effect. It is the reason why some of the currently developed PSS‐TE devices cannot achieve a continuous and stable 24 h electricity output. In contrast, the electricity output of Type 2 significantly increases in the daytime after adding the photothermal film at the hot end to increase the *T*_h_. However, the output performance shows a drop at night compared to Type 1. This is because the photothermal film with high absorptivity in the spectral range of 0–25 µm can exhibit efficient photothermal conversion in the daytime to maintain the temperature of the hot end but shows an obvious radiation‐cooling effect at night, dropping the Δ*T*. To avoid performance degradation at night, Type 3 with a selective photothermal effect in the hot end has been designed. It can exhibit excellent and stable electricity output over the whole day, indicating that the PSS‐TE devices have the potential to achieve 24 h continuous output through coupling the radiation cooling and selective photothermal conversion.

To further explore the influence of the spectral characteristics of radiation‐cooling film and selective photothermal film on the electrical performance of the PSS‐TE device, the short‐wave (0–8 µm) absorptivity *ε*_SW_ and long‐wave (8–25 µm) emissivity *ε*_LW_, as simplified parameters of ${\bar{\varepsilon }}_{\mathrm{solar}}$ and ${\bar{\varepsilon }}_{\mathrm{LWIR}}$, were employed as the variables in the COMSOL simulation (Figure 2D). As presented in Figure 2E and Figure S2A (Supporting Information), higher *V*_oc_ and *P*_max_ can be achieved if the *ε*_LW_ of the radiation‐cooling film and the *ε*_SW_ of the selective photothermal film are increased. When the *ε*_LW_ and *ε*_SW_ are small (for instance, 0.5, in Figure 2F), the influence of the solar irradiation density on the electrical performance of the PSS‐TE device is large. But when the *ε*_LW_ and *ε*_SW_ increase to about 1, the electrical performance of the PSS‐TE device is relatively stable under different solar irradiation intensities (Figure 2F and Figure S2B, Supporting Information). Therefore, high‐quality radiation cooling (high ${\bar{\varepsilon }}_{\mathrm{LWIR}}$) with a large ${\bar{R}}_{\mathrm{solar}}$ and selective photothermal absorption (high ${\bar{\varepsilon }}_{\mathrm{solar}}$) with a low ${\bar{\varepsilon }}_{\mathrm{LWIR}}$ can greatly improve the spectral utilization efficiency and thus be beneficial for the realization of the continuous and unipolar 24 h Δ*T*. These simulation results further clarify the relationship between the coupling of radiation‐cooling and photothermal effects and the electrical performance of TE devices, and contribute to guiding the practical experimental fabrication of PSS‐TE devices.

### 3.2 Selection of Radiation‐Cooling Films

In this section, the experimental realization of radiation‐cooling films with excellent performance, thereby reducing the cold‐end temperature of the PSS‐TE devices, will be introduced. Among the numerous radiation‐cooling materials that have high ${\bar{R}}_{\mathrm{solar}}$ and ${\bar{\varepsilon }}_{\mathrm{LWIR}}$, P(VdF‐HFP) film is chosen in this study due to its advantages of facile preparation and excellent radiation‐cooling effect. Dielectric microspheres or polymer microspheres, if added to radiation‐cooling materials, can regulate solar reflectivity by Mie scattering.^[^ ^49^, ^50^, ^51^ ^]^ Given these, P(VdF‐HFP) polymers with mixed Al_2_O_3_ dielectric microspheres were employed in this work. The influence of the thicknesses and Al_2_O_3_ dielectric microspheres on the spectral characteristics of radiation‐cooling films was then investigated.

**Figure 3:**
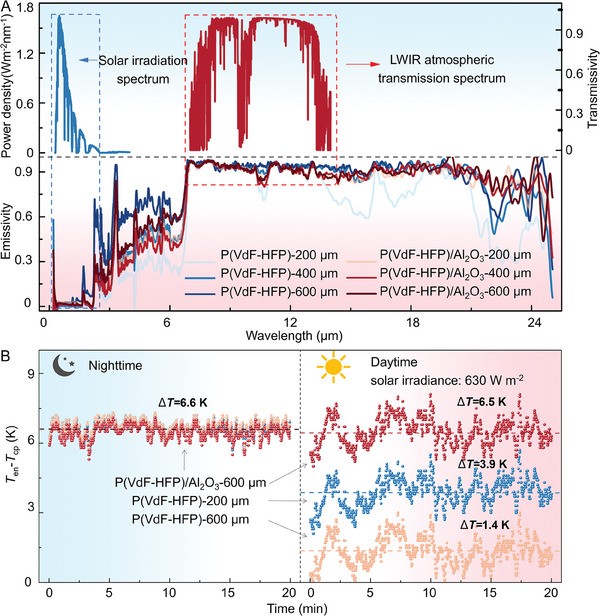
The performance of the synthesized radiation–cooling films. A) Solar irradiation spectrum, LWIR atmospheric transmission spectrum, and emission spectrum of radiation‐cooling films. B) The Δ*T* between the radiation‐cooling films and the environment at night and in the daytime, respectively.

**Figure** **3**A shows the emission spectrum of the synthesized P(VdF‐HFP) and P(VdF‐HFP)/Al_2_O_3_ films under different thicknesses, as well as the solar irradiation spectrum and atmospheric transmission spectrum. By observing the overlapping regions of the above spectra, these radiation‐cooling films exhibit good reflectivity in the solar spectrum and high emissivity in the atmospheric window. The ${\bar{R}}_{\mathrm{solar}}$ and ${\bar{\varepsilon }}_{\mathrm{LWIR}}$ for different radiation‐cooling films are summarized in Table S2 (Supporting Information). Among them, the ${\bar{R}}_{\mathrm{solar}}$ of P(VdF‐HFP) film with a thickness of 200 µm is 0.87, and the ${\bar{\varepsilon }}_{\mathrm{LWIR}}$ is 0.92. When the thickness of P(VdF‐HFP) film increases to 600 µm, the ${\bar{\varepsilon }}_{\mathrm{LWIR}}$ increases to 0.94. The addition of Al_2_O_3_ microspheres further improved the ${\bar{R}}_{\mathrm{solar}}$ of the film, reaching a maximum of 0.90. The transmission spectrum and reflection spectrum of radiation‐cooling films can be seen in Figure S3 (Supporting Information). Through comparative analysis of the above values, the P(VdF‐HFP)‐200 µm film, P(VdF‐HFP)‐600 µm film, and P(VdF‐HFP)/Al_2_O_3_‐600 µm film that have high ${\bar{R}}_{\mathrm{solar}}$ or ${\bar{\varepsilon }}_{\mathrm{LWIR}}$ were selected for subsequent cooling experiments. Large‐scale preparation of the radiation‐cooling films on copper substrates is successfully achieved through temperature control (Figure S4, Supporting Information), although its ${\bar{R}}_{\mathrm{solar}}$ and ${\bar{\varepsilon }}_{\mathrm{LWIR}}$ are lower than that reported by Yang et al.^32^ Moreover, the radiation‐cooling films are tightly attached to the substrate, which accelerates the transfer of heat and improves the radiation‐cooling effect.

The radiation‐cooling films deposited on the copper plates were placed in the foam boxes and then sealed with Polyethylene (PE) films. By comparing the copper plate temperature (*T*_cp_) and the environmental temperature (*T*_en_), the actual cooling performance of different radiation‐cooling films can be evaluated. Figure 3B shows that all three radiation‐cooling films exhibit excellent cooling effects at night, which can exhibit a temperature that is about 6.6 K lower than that of the environmental temperature. However, in the daytime, these films exhibit significantly distinct performance, in which the P(VdF‐HFP)/Al_2_O_3_‐600 µm film shows the largest average Δ*T* of about 6.5 K, due to its higher ${\bar{R}}_{\mathrm{solar}}$ (Table S2, Supporting Information) that helps to reduce the heat deposition. These results suggest that the P(VdF‐HFP)/Al_2_O_3_‐600 µm film could show a good radiation‐cooling effect both in the daytime and at night, which was selected for forming the cold end during the subsequent preparation of PSS‐TE devices.

### 3.3 Selective Photothermal Films

**Figure 4:**
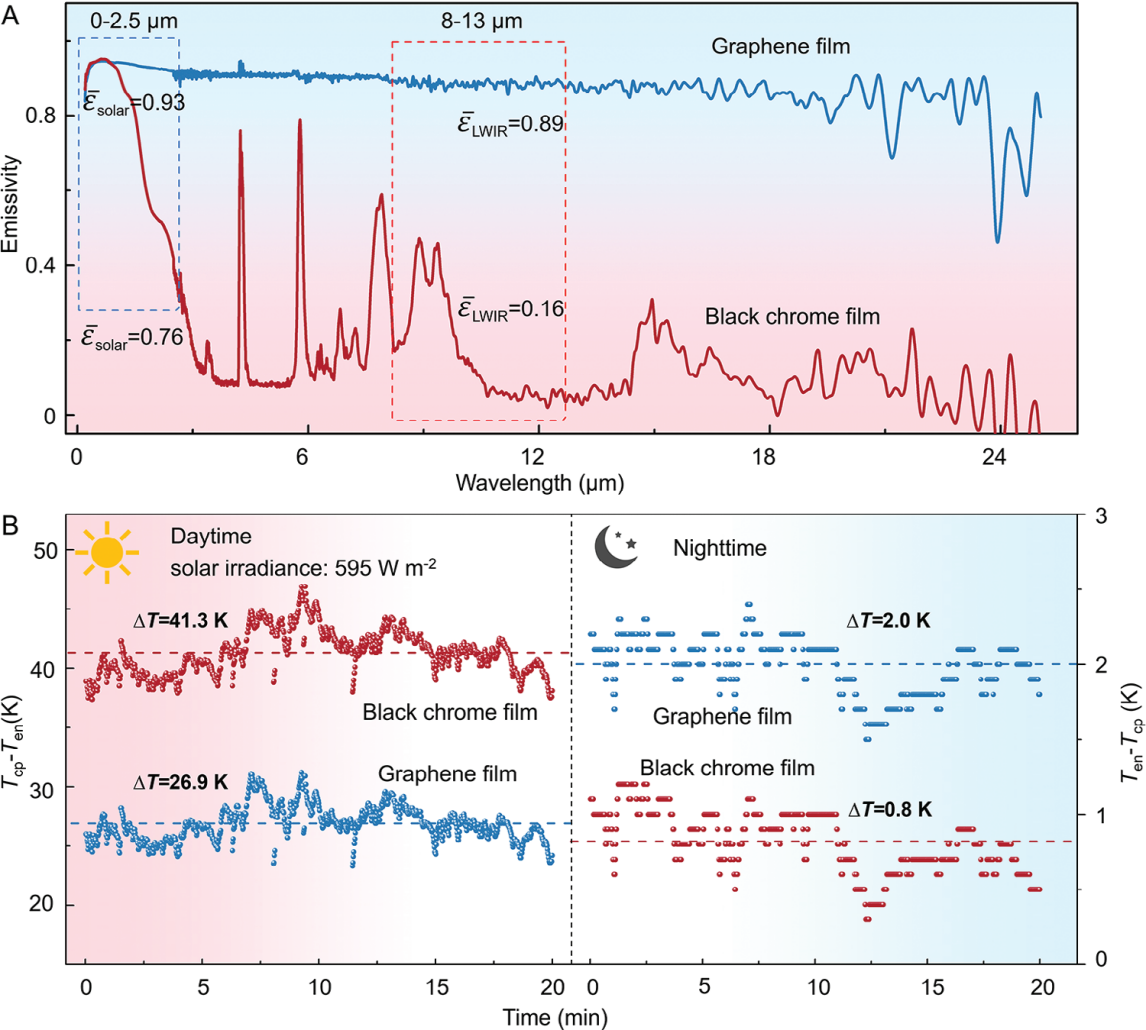
The photothermal effect of the graphene and black chrome films. A) The emission spectrum. B) The Δ*T* between the copper plates (coated with black chrome film and graphene film, respectively) and environmental temperature at night and in the daytime.

To improve the hot‐end temperature of the PSS‐TE devices, the spectral properties of photothermal materials were analyzed. To maintain the 24 h continuous and unipolar Δ*T*, the photothermal unit on the hot end is required to absorb the sunlight as much as possible in the daytime while reducing its radiation cooling (or thermal radiation) at night. Therefore, it is important to seek a suitable material with selective heat absorption/emission. The Graphene film, a well‐known photothermal material,^[^ ^52^ ^]^ was first studied. The blue line in **Figure** **4**A shows the absorption/emission spectrum of graphene film. The prepared graphene film has high emissivity in the whole spectrum range, which means that the material can absorb the sunlight well for its high ${\bar{\varepsilon }}_{\mathrm{solar}}$, and accumulate heat to become the hot end in the daytime. Simultaneously, it has a high radiation‐cooling effect at night for its high ${\bar{\varepsilon }}_{\mathrm{LWIR}}$, which would cause the lower temperature of the photothermal film compared with the environmental temperature and reduce the Δ*T* between the hot and cold ends of the PSS‐TE device at night. Given this, the black chrome film, as an excellent representative of selective photothermal materials,^[^ ^53^ ^]^ comes into our sight. As shown in Figure 4A, the black chrome purchased from Dezhou KNEAR Co., Ltd. has high emissivity in the spectrum of 0–2.5 µm, which can effectively absorb sunlight and convert it into heat. Subsequently, the emissivity decreases rapidly with the increase of wavelength (>2.5 µm). Its average emissivity is only 0.16 in the range of 8–13 µm, which is far lower than that of 0.89 for the graphene film. This indicates that the black chrome film will not generate a significant temperature drop of the hot end in the night owing to its weak radiation‐cooling effect. The transmission spectrum and reflection spectrum of the graphene and black chrome films are shown in Figure S5 (Supporting Information).

The following experiments were then carried out to examine the photothermal effect of the graphene and black chrome films during the day and at night. The photothermal films deposited on the copper plates are placed in the foam boxes and then sealed with PE films. The performance of the two films in the daytime and at night is evaluated by comparing the *T*_cp_ and the *T*_en_ in Figure 4B. In the daytime, both the graphene and black chrome films lead to increased temperatures in the copper plates. Compared to the graphene film with a Δ*T* of 26.9 K, black chrome film obtained a larger Δ*T* of 41.3 K. This is because the black chromium film can effectively reduce thermal radiation due to its small ${\bar{\varepsilon }}_{\mathrm{LWIR}}$, thereby accumulating more heat and leading to a higher Δ*T*. Similarly, at night, the low ${\bar{\varepsilon }}_{\mathrm{LWIR}}$ of the black chromium film generated a weak radiation‐cooling effect, and the temperature of the black chromium film is only 0.8 K lower than the environmental temperature, while the temperature of graphene film is 2 K lower. Therefore, the black chromium film is selected as the preferred photothermal material for forming the hot end during the subsequent preparation of PSS‐TE devices due to its ability to maintain high temperatures throughout the whole day.

### 3.4 Proof‐of‐Concept of PSS‐TE Device

**Figure 5:**
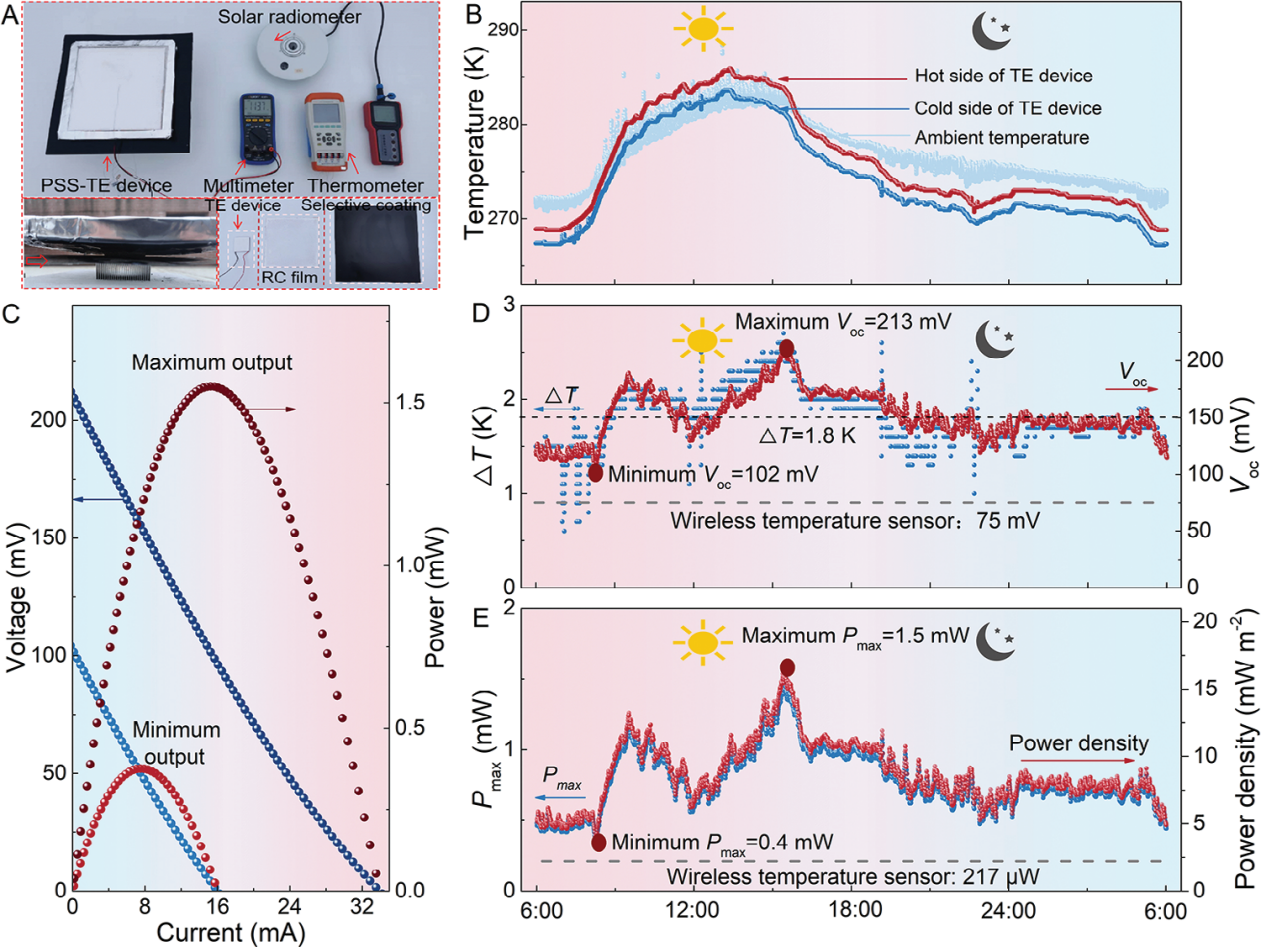
The output performance of the PSS‐TE device during a 24 h test. A) The optical image of the PSS‐TE device and test equipment. B) Temperature of the hot and cold end of the device, and environmental temperature throughout the day. C) The *V*–*I*/*P*–*I* curve of minimum and maximum output of the PSS‐TE device. D) The variation curve of Δ*T* and *V*_oc_. E) The variation curve of *P*_max_ and power density.

Based on the coupling effect of radiation cooling and selective photothermal absorption on the spectrum (0–2.5 µm and 8–13 µm), a prototype PSS‐TE device was finally successfully assembled, by coating the P(VdF‐HFP)/Al_2_O_3_‐600 µm radiation‐cooling film and the black chrome photothermal film on the cold end and hot end, respectively. As shown in **Figure** **5**A, the interfaces between the TE device, radiation‐cooling film, and selective photothermal film were connected using the high thermal‐conductivity silicone grease. The size of the built‐in TE device, radiation‐cooling film, and selective photothermal film was 55 × 55 mm^2^, 200 × 200 mm^2^, and 300 × 300 mm^2^, respectively. To examine the performance of the PSS‐TE device, it was placed on a spacious roof and tested for a whole day. Figure 5B shows the temperature curve of the hot and cold ends of the PRC‐TE device for the whole day. The environmental temperature fluctuates between 271.3 and 284.4 K, and the maximum solar radiation intensity reaches about 600 W m^−2^ (Figure S6, Supporting Information).

Although the environmental temperature fluctuated significantly throughout the day, the temperature of the hot end was always higher than that of the cold end for the PSS‐TE device, ensuring a unipolar Δ*T* for the whole day. Figure 5C shows the *V*–*I*/*P*–*I* curve corresponding to the maximum or minimum values obtained by the PRC‐TE device for the whole day. The time‐dependent Δ*T*, *V*_oc_, *P*_max_ and power density are comprehensively shown in Figure 5D,E. Throughout the day, the average Δ*T* between the two ends remains at about 1.8 K in Figure 5D, which leads to high electricity output in the whole day. The changing trend of the *V*_oc_ of the PSS‐TE device is consistent with the varied Δ*T*, which is always unipolar and higher than 102.6 mV. The prerequisite for an electronic device to maintain a continuous operation for 24 h is that a stable power supply can be always provided. Therefore, the minimum *P*_max_ can be used as a metric to gauge the electrical performance of the developed 24 h self‐sustained devices. The minimum *P*_max_ of the PSS‐TE device is 0.4 mW and the normalized power density based on the area of the selective photothermal film remains above 4.4 mW m^−2^ throughout the day (Figure 5E), which is currently the highest value for a 24 h PSS‐TE device (Table 1). In addition, the maximum *P*_max_ reaches about 1.5 mW and the corresponding normalized power density is as large as 16.8 mW m^−2^. The developed PSS‐TE device produces continuous unipolar Δ*T* and electricity output throughout the day, significantly extending the run time of PSS‐TE devices from night to all day.

**Table 1:** Comparison of the currently self‐sustained TE devices (Note: “+ and ‐” represent the output polarity of the PSS‐TE device. Once the *P*_min_ of the PSS‐TE device approaches 0, the device loses the possibility of powering electronic devices continuously).

Night operation	Daytime operation	24 h Power supply	Minimum *P*_max_	Corresponding power density	Application demonstration	Sources
Yes; +	Yes; +	Yes	0.4 mW	4.4 mW m^−2^	Yes	This work
Yes; +	Yes; +	Yes	0.03 mW	3.85 mW m^−2^	No	[55]
Yes; +	No	No	0.4 mW at night	12.7 mW m^−2^ at night	Yes	[34]
Yes; ‐	Yes; +	No	0	0	No	[43]
Yes; +	Yes; +	No	0	0	No	[48]
Yes; +	Yes; +	No	–	0.3 mW m^−2^	No	[54]

It is gratifying that the electrical performance of the PSS‐TE device can be further improved by optimizing the TE device, radiation‐cooling material, and selective photothermal material. Given the demonstrated electricity output, the developed PSS‐TE device is expected to provide a 24 h power supply for low‐power IoT nodes.

### 3.5 24 h Powering of the IoT Node and LED Lighting

**Figure 6:**
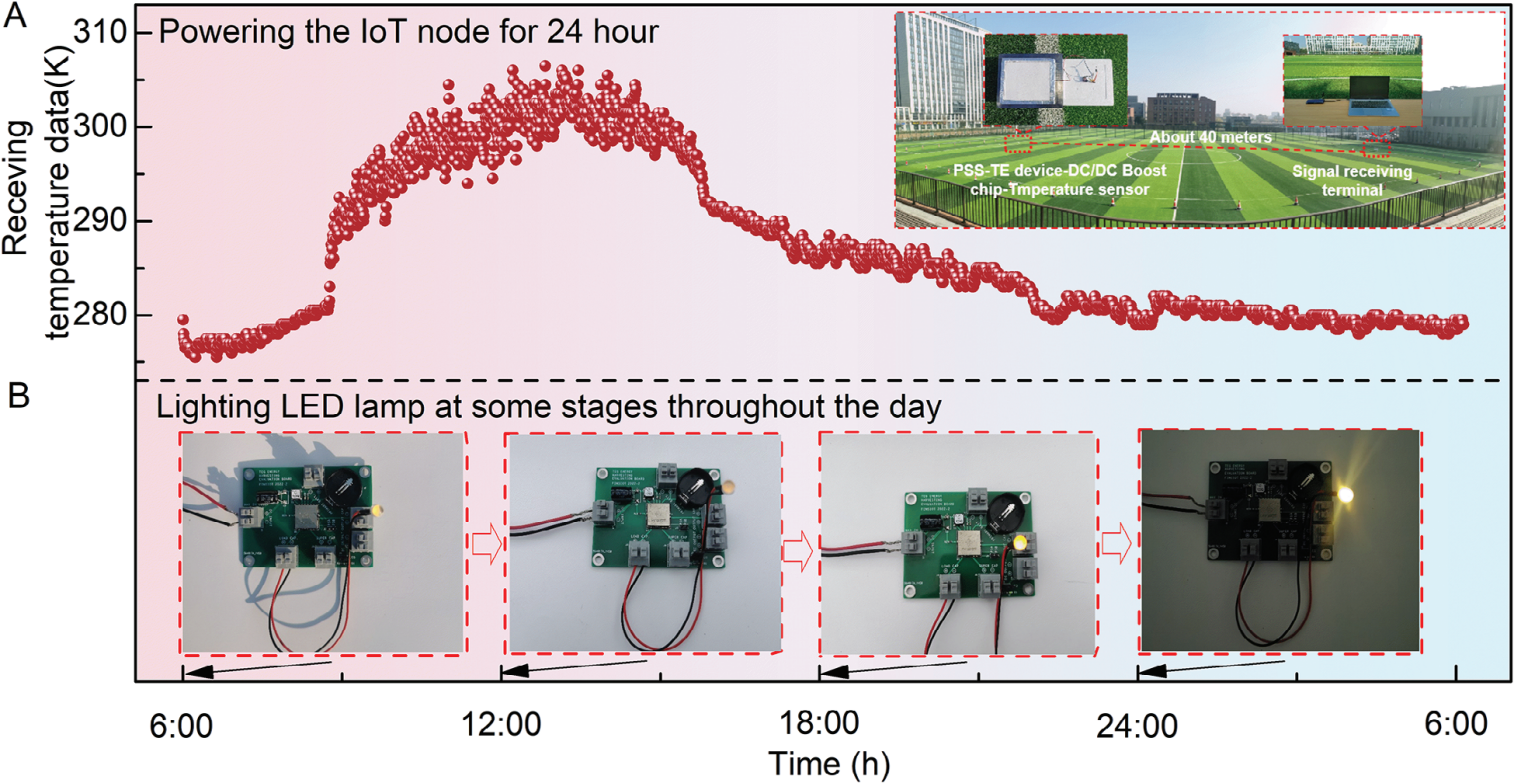
Application demonstration of the developed PSS‐TE devices. A) The 24 h wireless temperature transmission. B) LED Lighting at 6:00, 12:00, 18:00, and 24:00.

The electricity output generated by the PSS‐TE device can be successfully utilized to support the real‐time working of IoT nodes, demonstrated using the wireless temperature sensor. As exhibited in Figure S7 (Supporting Information), the wireless temperature sensor powered by the PSS‐TE device can transmit data over a long distance of about 40 meters on the playground. First, the output voltage generated by the PSS‐TE device was raised to 3 V through the DC/DC boost chip to meet the requirement of the start‐up voltage for the wireless temperature sensor and transmission module. After that, the signal sent by the wireless temperature sensor reaches the receiving module through wireless transmission and then is converted into temperature data after processing. In our preliminary attempts, we found that only when the *V*_oc_ and *P*_max_ of the PSS‐TE device are higher than 75 mV and 217 µW, the wireless temperature sensor could start to work. Figure 5D,E shows that the electricity output of the PSS‐TE device is much higher than the start‐up value of the temperature sensor in the whole day, which means that the developed PSS‐TE device can power wireless temperature sensors to continuously work for 24 h, as verified in **Figure** **6**A. The wireless temperature sensors transmit approximately 2880 data points throughout the day (with one data sent in approximately every 30 seconds), providing an extensive level of data collection. As a more intuitive display, the PSS‐TE device successfully lights up the LED throughout the day (Figure 6B and Figure S8, Supporting Information). These results demonstrate the feasibility of PSS‐TE devices for providing off‐grid power supply to IoT nodes and LED lighting for a whole day, indicating their promising potential for power generation across various other fields.

## 4 Conclusion

In summary, a self‐sustained power supply device that can achieve a 24 h steady electricity output has been developed by integrating TE technology, the radiation‐cooling effect, and selective photothermal conversion. With the COMSOL simulation, the coupling between the radiation‐cooling and the selective photothermal effect is found to be the key to maintaining the 24 h continuous unipolar Δ*T* and electricity output for the PSS‐TE device. To improve the Δ*T* of the PSS‐TE device, radiation‐cooling film of the 600 µm thickness P(VdF‐HFP)/Al_2_O_3_ and selective photothermal film of black chromium are selected through spectral analysis, as well as the practical experiments. The fabricated PSS‐TE device generates an average Δ*T* of about 1.8 K, leading to an electricity output higher than 102 mV and 0.4 mW during the whole day. Remarkably, the PSS‐TE device can successfully support the wireless temperature sensor and the LED to operate continuously for 24 h. These results highlight the application prospects of self‐sustained TE technology for power generation in various fields.

## 5 Experimental Section

### Preparation of Radiation‐Cooling Films

Poly(vinylidene fluoride‐co‐hexafluoropropene) (P(VdF‐HFP)) powders of 10 g from Dongguan Zhanyang Polymer Materials Co., Ltd. was added into 60 mL acetone (AC), and the solution was stirred magnetically at room temperature for 1 h. 20 mL acetone was mixed with 10 mL deionized water, and then the mixed solution was dropped into the acetone solution of P(VdF‐HFP). The process of magnetic stirring lasted for 2 h until the mixed acetone solution of P(VdF‐HFP) was transparent. The 1 µm size Al_2_O_3_ microspheres and the P(VdF‐HFP) solution were mixed in a mass ratio of 1:50 for comparison. The mixed P(VdF‐HFP) solution was poured onto the cleaned copper substrate. The rapid evaporation of acetone led to the separation of P(VdF‐HFP) and water phase, forming huge amounts of microdroplets and nanodroplets. The water gradually evaporated and the P(VdF‐HFP) film was formed in about 24 h. The micropores and nanopores in the film effectively backscattered sunlight and enhanced thermal emission. The thickness of the film deposition was controlled by regulating the quality of the solution. There is always a part of the radiation‐cooling film that detaches from the substrate, which is often encountered during the film process. To overcome the demolding phenomenon, this experiment took an electric heating table to provide a balanced temperature (303 K), enabling the preparation of a large area, strong‐adhesion radiation‐cooling film (Figure S4, Supporting Information).

### Preparation of Photothermal Films

The graphene coating purchased from 2DGr Taobao merchant was brushed on the clean aluminum plate. After 5 h of standing treatment, the coating will form a dense black film. The black chrome film was purchased from Dezhou KNEAR New Materials Technology Co., Ltd. The chromium film was deposited on the surface of the substrate layer by layer in the form of a single‐atom film, and at the same time, it was used as an absorbent and a mirror agent to ensure high solar absorptivity and low long‐wave emissivity.

### Temperature Testing Experiment

A 10 × 10 cm^2^ radiation‐cooling or selective photothermal unit (with a copper substrate) was placed inside appropriately sized foam boxes and securely sealed with PE film. The K‐type thermocouple probe from KAIPUSEN is then positioned beneath the radiation‐cooling or selective photothermal unit to record *T*_cp_. Meanwhile, another K‐type thermocouple probe is exposed to the ambient air to measure the environmental temperature *T*_en_. By comparing the temperature difference between these two positions utilizing the JINKO JK808 temperature tester, the efficacy of the radiation‐cooling and the selective photothermal film can be assessed.

### Assembly and Test of the PSS‐TE Device

The PSS‐TE device consisted of a radiation‐cooling unit, a TE device, and a selective photothermal unit. The radiation‐cooling unit was a 200 × 200 mm^2^ radiation‐cooling film deposited on the copper plate. The TE device (TEG‐241‐1.4‐2) was purchased from Fu Electronics Store. It has 241 pairs of TE legs with a size of 55 × 55 mm^2^ and the internal resistance is tested to be 6.3 Ω. The selective photothermal unit includes a 300 × 300 mm^2^ selective photothermal film and an aluminum sink. The radiation‐cooling unit was put on the upper end of the TE device. Both were placed in the foam box and sealed with PE film. The bottom foam was removed to ensure that the TE device could contact the photothermal unit. The structure of the whole PSS‐TE device is shown in Figure 1A. The prepared PSS‐TE was placed on a spacious roof for testing. The *V*_oc_ of the device was recorded by a multimeter, and the *P*_max_ was calculated through the formula of *P*_max_ = *V*_oc_^2^ (4*R*)^−1^. The thermometers were used to record the temperature of the hot and cold ends of TE devices and the environmental temperature. At the same time, the solar radiometer was used to record the solar irradiance intensity for the whole day. The *V*–*I*/*P*–*I* curve of the device was obtained with the Keithley 2400.

### Working Principle of Wireless Temperature Sensor

The PSS‐TE device was directly connected to the DC/DC boost chip (LTC‐3108), which increased the voltage output from 100 mV to 3 V. When this output met the voltage and power requirements of the transmission module (APC300) and temperature sensor (DS18B20), the transmission module would transmit the collected temperature data. At this time, the remote receiving module (APC250S) received the data signals and finally stored them on the computer through software processing. Ideally, the transmission distance between the transmitter and receiver can be up to 700 m. This experiment simulated the whole process of data acquisition, transmission, reception, and display of passive IoT nodes.

## Conflict of Interest

The authors declare no conflict of interest.

## Supporting information

Supporting Information

Supplemental Video 1

Supplemental Video 2

## Data Availability

The data that support the findings of this study are available from the corresponding author upon reasonable request.
