# Supplementary material for: Passive Self‐Sustained Thermoelectric Devices Powering the 24 h Wireless Transmission via Radiation‐Cooling and Selective Photothermal Conversion
Source: Adv Sci (Weinh). 2024 Apr 4;11(23):2309871. doi: 10.1002/advs.202309871 (PMC11186140; doi:10.1002/advs.202309871)
Supplement: Supplementary file 1 — Supporting Information [file ADVS-11-2309871-s003.pdf]

ADVANCED SCIENCE Open Access

Supporting Information

for *Adv. Sci.*, DOI 10.1002/advs.202309871

Passive Self-Sustained Thermoelectric Devices Powering the 24 h Wireless Transmission via Radiation-Cooling and Selective Photothermal Conversion

Kai Liu, Yaoguang Ma, Yuzheng Li, Yunxiao Wu, Chenguang Fu and Tiejun Zhu**

Supporting Information

Passive self-sustained thermoelectric devices powering the 24-hour wireless transmission via radiation-cooling and selective photothermal conversion

Kai Liu, [a,b] *Yaoguang Ma,*[c,d] *Yuzheng Li,* [a] *Yunxiao Wu,*[a] *Chenguang Fu,*[*, a,b] *and Tiejun Zhu*[*, a,b]

Supplementary Note

Note S1. Working principle for the PSS-TE devices

First, the internal mechanism of the radiation-cooling effect is introduced. At night, the net radiant cooling capacity P_{net} of the radiation cooling film can be expressed as the difference between the energy radiated outward (P_{rad}) and the energy received from the environment (P_{en}):

$$P_{\text{net}} = P_{\text{rad}} - P_{\text{en}}$$

$$= A_c \int d\Omega \cos\theta \int_0^\infty d\lambda\, I_{BB}(T_c,\lambda)\varepsilon(\lambda,\theta) - A_c \int d\Omega \cos\theta \int_0^\infty d\lambda\, I_{BB}(T_{en},\lambda)\varepsilon(\lambda,\theta)\varepsilon_{atm}(\lambda,\theta)$$

$$= A_c \int d\Omega \cos\theta \int_0^\infty d\lambda\, I_{BB}(T_c,\lambda)\varepsilon(\lambda,\theta)[1-\varepsilon_{en}(\lambda,\theta)] \quad (\text{while } T_c = T_{en}) \qquad (1)$$

where A_c is the area of radiation cooling film, I_{bb} is the spectral intensity, ε is the material emissivity, and ε_{atm} is the atmospheric emissivity. It can be seen from the above formula that when $\varepsilon(\lambda,\theta)$ [1- $\varepsilon_{atm}(\lambda,\theta)$] is large, a large P_{net} can be obtained. That is, to maximally exert the role of the radiation-cooling effect, the emission spectrum of the material should match the atmospheric transmission spectrum.

In the daytime, it is necessary to consider the solar radiation. In this condition, the P_{net} is:

$$P_{\text{net}} = P_{\text{rad}} - P_{\text{en}} - P_{\text{sun}}$$

$$= A_c \int d\Omega \cos\theta \int_0^\infty d\lambda\, I_{BB}(T_c,\lambda)\varepsilon(\lambda,\theta)[1-\varepsilon_{atm}(\lambda,\theta)] - \int_0^{2.5} d\lambda\, I_{solar}(\lambda)\varepsilon(\lambda,\theta) \qquad (2)$$

It can be seen that the emission spectrum of the material not only needs to match with the atmospheric window but also should have low emissivity in the solar spectral range in daytime conditions.

Based on these, the theoretical model of the PRC-TE device was further established. As shown in Fig. S1, solar irradiation energy is used as the energy source at the hot end of the TE

device, and the cold end emits heat through the radiation-cooling effect. The energy balance at both ends is shown in the following formula:

Hot end : $P_{\text{net,h}} = S_{pn}T_hI + K(T_h - T_c) - \frac{1}{2}I^2R$ (3)

Cold end : $P_{\text{net,c}} = S_{pn}T_cI + K(T_h - T_c) + \frac{1}{2}I^2R$ (4)

The $P_{\text{net,c}}$ of the cold end can be expressed as:

$$P_{\text{net,c}} = P_{\text{rad,c}} - P_{\text{en,c}} - P_{\text{sun,c}}$$

$$= A_c \int d\Omega \cos\theta \int_0^\infty d\lambda\, I_{BB}(T_c,\lambda)\varepsilon(\lambda,\theta)[1-\varepsilon_{atm}(\lambda,\theta)] - A_c \int_0^{2.5} d\lambda\, I_{solar}(\lambda)\varepsilon(\lambda,\theta) \quad (5)$$

The $P_{\text{net,h}}$ of hot end can be expressed as:

$$P_{\text{net,h}} = P_{\text{sun,h}} - P_{\text{rad,h}} - P_{\text{en,h}}$$

$$= A_h \int_0^{2.5} d\lambda\, I_{solar}(\lambda)\varepsilon(\lambda,\theta) - A_h \int d\Omega \cos\theta \int_0^\infty d\lambda\, I_{BB}(T_h,\lambda)\varepsilon(\lambda,\theta)[1+\varepsilon_{atm}(\lambda,\theta)] \quad (6)$$

The current I is:

$$I = \frac{S_{pn}(T_h - T_c)}{R + r} \quad (7)$$

where A_h are the areas of the hot end, T_h and T_c are the temperatures of the hot and cold end, respectively, and the S, K, and R are the Seebeck coefficient, the thermal conductance, and the electrical resistance of the TE device.

When the internal resistance r of the external equipment is equal to the internal resistance R of the thermoelectric device, the maximum output power can be obtained. T_h and T_c, as well as the open-circuit valtage V_{oc} and the maximum output power P_{max} of the device, can be solved by combining the Equations. (3-7) and substituting the spectral characteristics, thermoelectric parameters, dimensions, and environmental factors of specific materials.

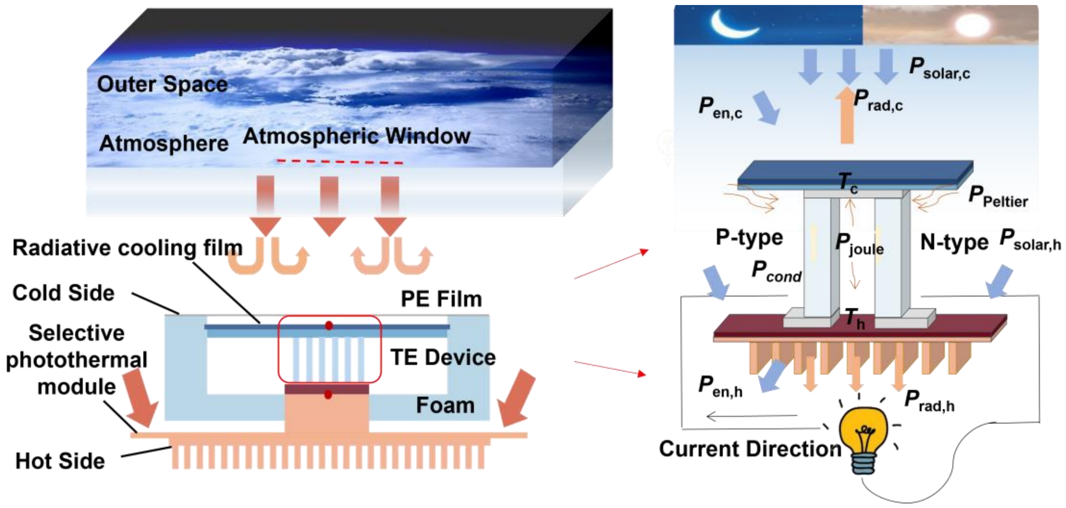

Figure S1. The schematic diagram of the energy conversion of the PSS-TE device.

Note S2. Parameters used in the COMSOL simulation

The dimensional parameters and material properties involved in the COMSOL simulation process are shown in Table. S1. The Seebeck coefficient, thermal conductivity, and electrical conductivity of TE materials are temperature-dependent. Therefore, the temperature-dependent curves were introduced in COMSOL simulation by interpolation to obtain a more reliable evaluation of the PSS-TE device. The atmospheric transmission spectrum data used are shown in Fig. 4a. In the simulation of Figure 2A-2D, the emission spectrum of the radiation-cooling films was set with the solar absorptivity$\bar{\varepsilon}_{\mathrm{solar}}$of 0.15 and the long-wave infrared emissivity or absorptivity $\bar{\varepsilon}_{\mathrm{LWIR}}$ of 0.9.

Table S1. Dimensional parameters and material properties involved in the simulation process.

**Parameters**	**Values**	**Source**
The size of the TE Leg	1.4×1.4×2 mm^3	Measured
The number of the TE Leg	242 pair	Measured
The area of radiation-cooling film	200×200 mm^2	Measured
The area of selective photothermal film	300×300 mm^2	Measured
Seebeck coefficient of P-type TE materials	217~239 μV K^{-1}	Measured
Seebeck coefficient of N-type TE materials	-210~-185μV K^{-1}	Measured
Thermal conductivity of P-type TE material	1.2~1.5 W m^{-1} K^{-1}	Measured
Thermal conductivity of N-type TE material	1.39~1.9 W m^{-1} K^{-1}	Measured
Electrical conductivity of P-type TE material	(0.46~0.95)×10^5 S m^{-1}	Measured
Electrical conductivity of N-type TE material	(0.59~1.0)×10^5 S m^{-1}	Measured
Thermal conductivity of copper	400 W m^{-1} K^{-1}	COMSOL

Heat capacity at constant pressure of copper	385 J kg^{-1}·K^{-1}	COMSOL
Thermal conductivity of Al_2O_3 ceramic	27 W m^{-1} K^{-1}	COMSOL
Thermal conductivity of foam	0.025-0.037 W m^{-1} K^{-1}	COMSOL
Ambient temperature	293 K	——

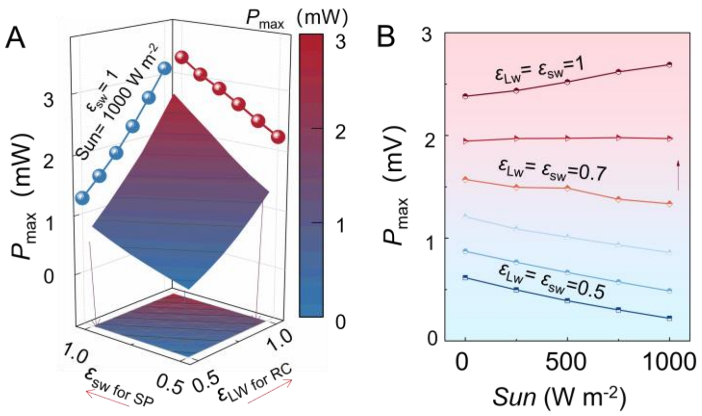

Figure S2. COMSOL simulation analysis. A) The mapped P_{max} under different ε_{LW} of the radiation-cooling film and ε_{sw} of the selective photothermal film. B) The mapped P_{max} under different solar radiation density.

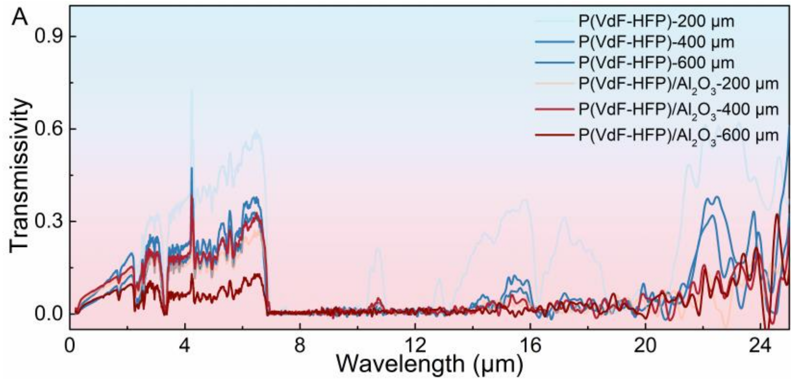

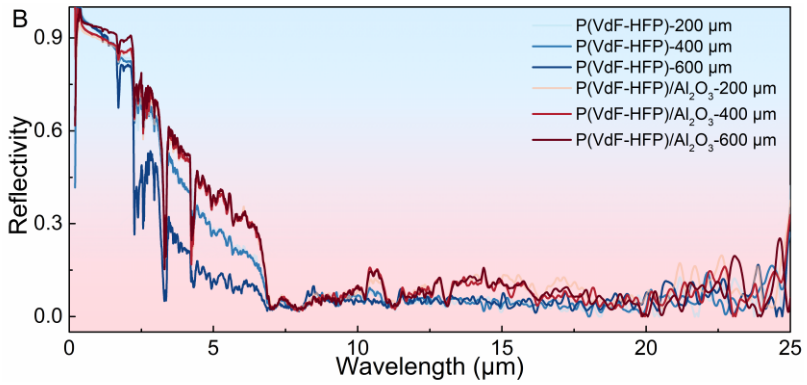

Figure S3. The spectral performance of radiation-cooling films. A) Transmission spectrum. B) Reflection spectrum.

Table S2. The spectral characteristics of various radiation-cooling films.

**Radiation-cooling film**	$\overline{R}_{\mathrm{solar}}$ **(0.3-2.5 μm)**	$\bar{\varepsilon}_{\mathrm{LWIR}}$ **(8-13 μm)**
P(VdF-HFP)-200 μm	0.87	0.92
P(VdF-HFP)-400 μm	0.87	0.94
P(VdF-HFP)-600 μm	0.82	0.94
P(VdF-HFP)/Al_2O_3-200 μm	0.86	0.92
P(VdF-HFP)/Al_2O_3-400 μm	0.87	0.91
P(VdF-HFP)/Al_2O_3-600 μm	0.90	0.92

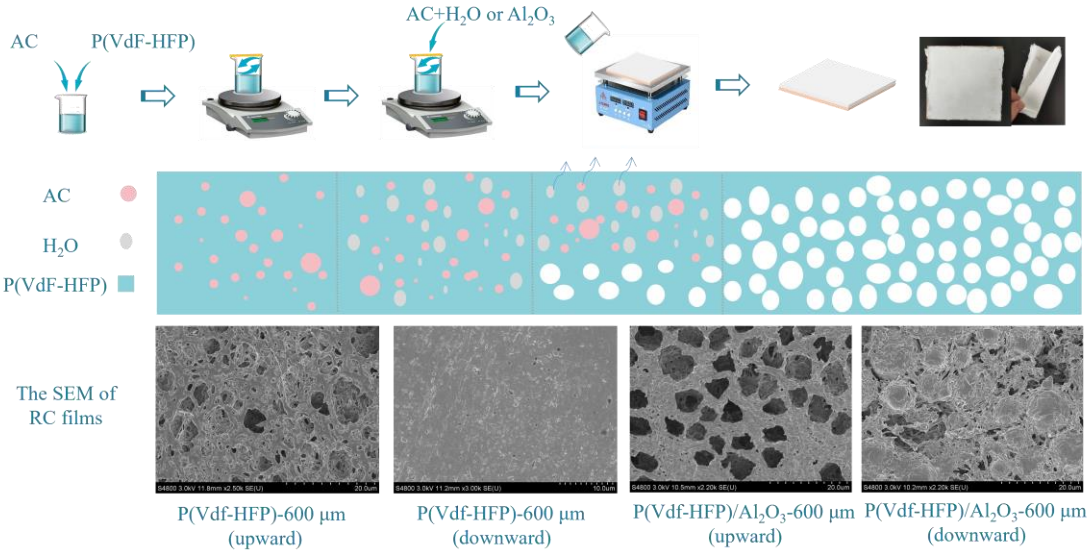

Figure S4. The preparation process of radiation-cooling films.

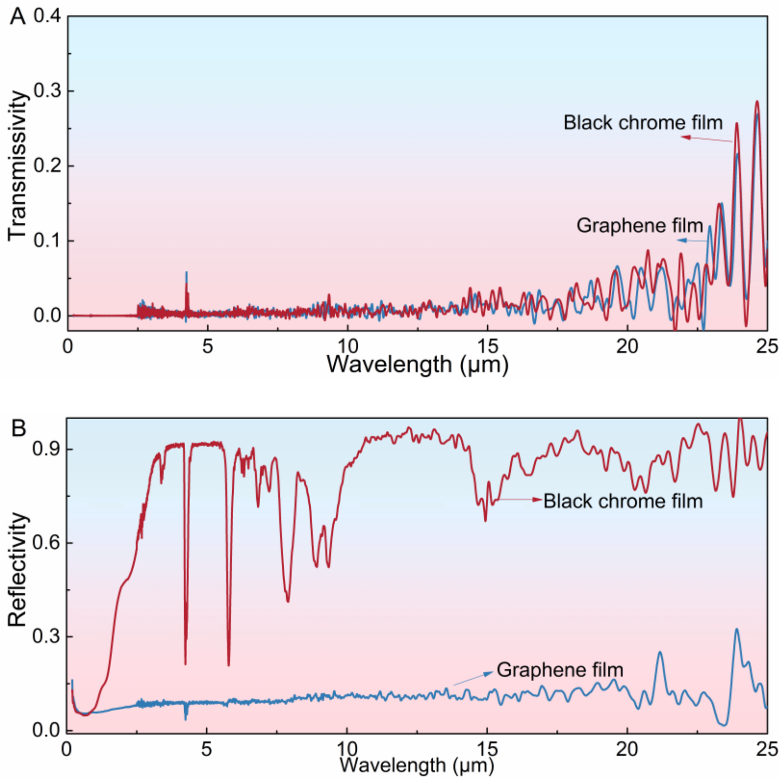

Figure S5. The spectral performance of the graphene and black chrome films. A) Transmission spectrum. B) Reflection spectrum.

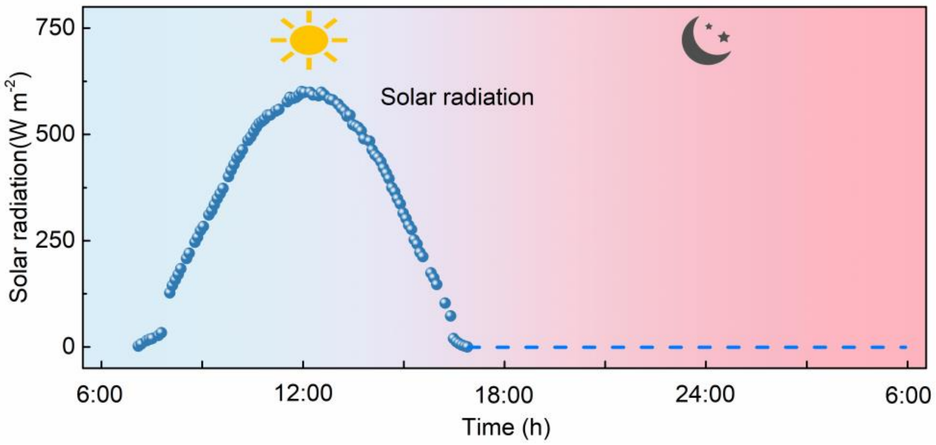

Figure S6. The solar radiation intensity throughout the day for the test.

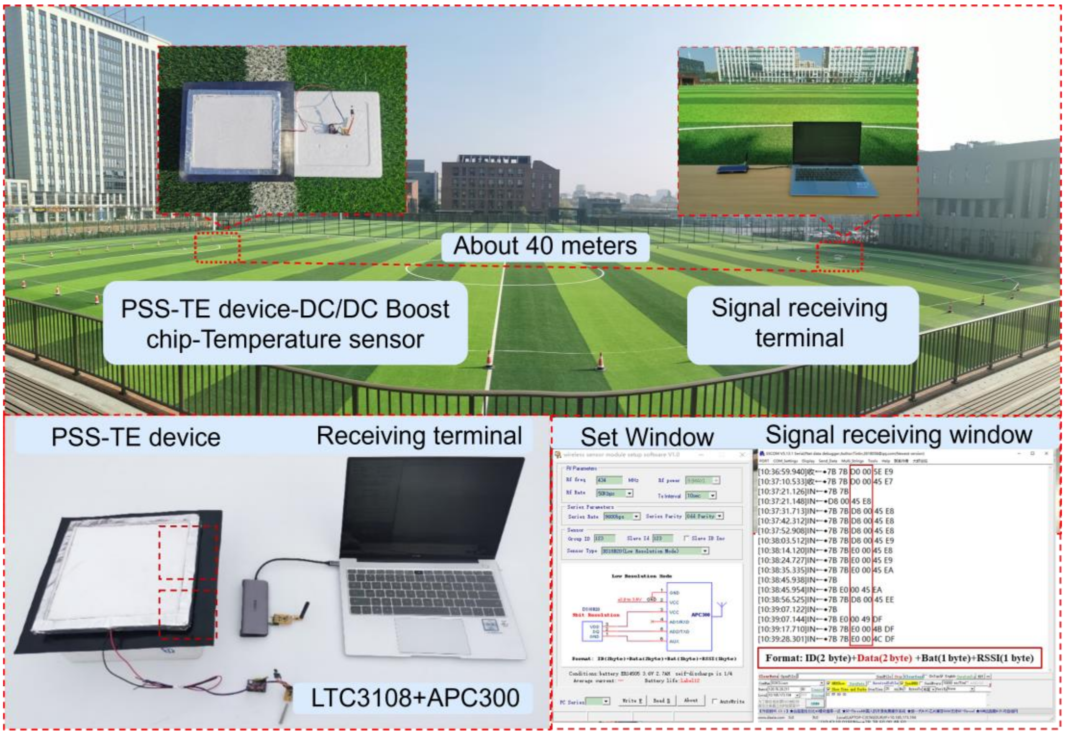

Figure S7. The test diagrams for the PSS-TE device supplying power to wireless temperature sensors.

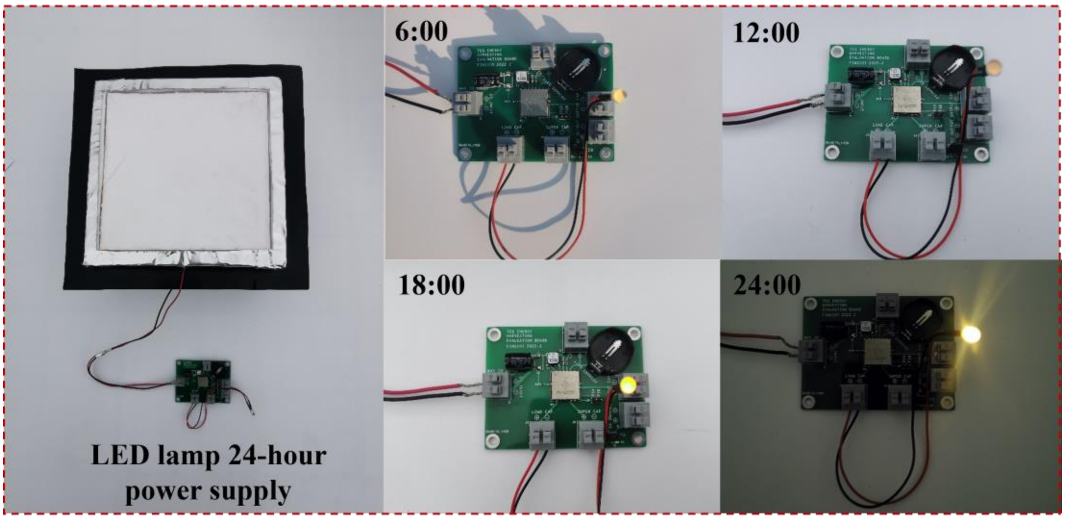

Figure S8. The test diagrams for the PSS-TE device supplying power to the LED.
